# Supplementary material for: A New 4-Thiazolidinone Derivative (Les-6490) as a Gut Microbiota Modulator: Antimicrobial and Prebiotic Perspectives
Source: Antibiotics (Basel). 2024 Mar 22;13(4):291. doi: 10.3390/antibiotics13040291 (PMC11047727; doi:10.3390/antibiotics13040291)
Supplement: Supplementary file 1 [file antibiotics-13-00291-s001.zip › antibiotics-2897908-supplementary.pdf]

Supplementary information

Supplementary Materials: Figures S1–S3: ^{1}H, ^{13}C, and LC–MS spectra of Compound **iii** (Les-6490). Figure S4. Comparative Taxonomic Relative Abundance at the Phylum Level in Different Groups (K, A, AL, L). Figure S5. Taxonomic Abundance Cluster Heatmap at Cluster′ C′. Figure S6. Biodiversity Curve Across Different Study Groups. Figure S7. Box Plot of the Difference in Observed Species Across Study Groups. Figure S8. Box Plot of Phylogenetic Diversity (PD) Whole Tree Across Groups. Figure S9. Box Plot Based on Unweighted UniFrac Distance Across Groups. Figure S10. Non-Metric Multidimensional Scaling (NMDS) Analysis of the 4 Groups. Figure S11. Between-group T-test Analysis (Orders *Coriobacteriales*, *Aeromonadales*). Figure S12. Between-group T-test Analysis (Families *Neisseriaceae*, *Coriobacteriaceae*, *Succivibrionaceae*). Figure S13. Photos of Rat Paws at Different Experimental Stages.

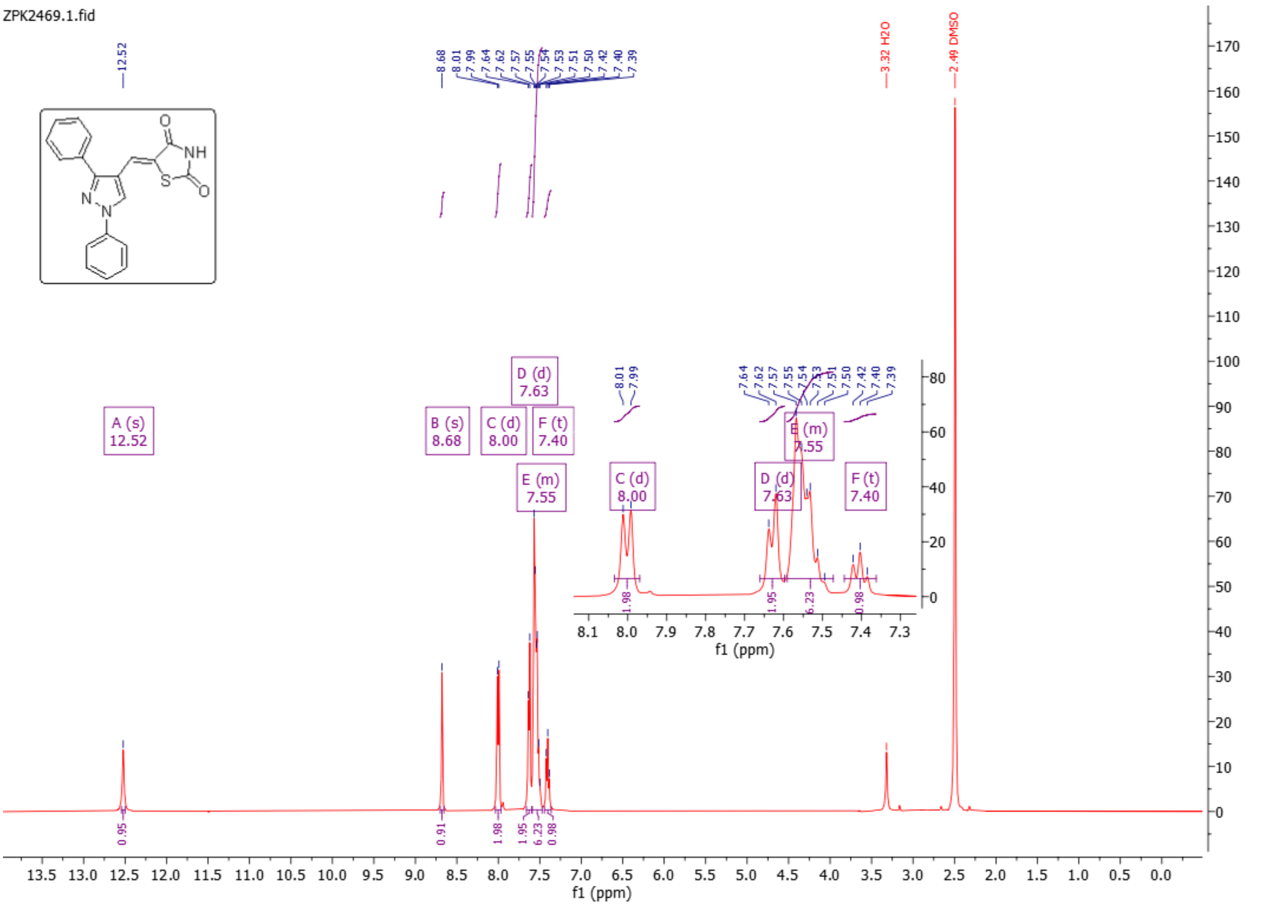

Figure S1. ^{1}H NMR spectrum of compound **iii** (Les-6490).

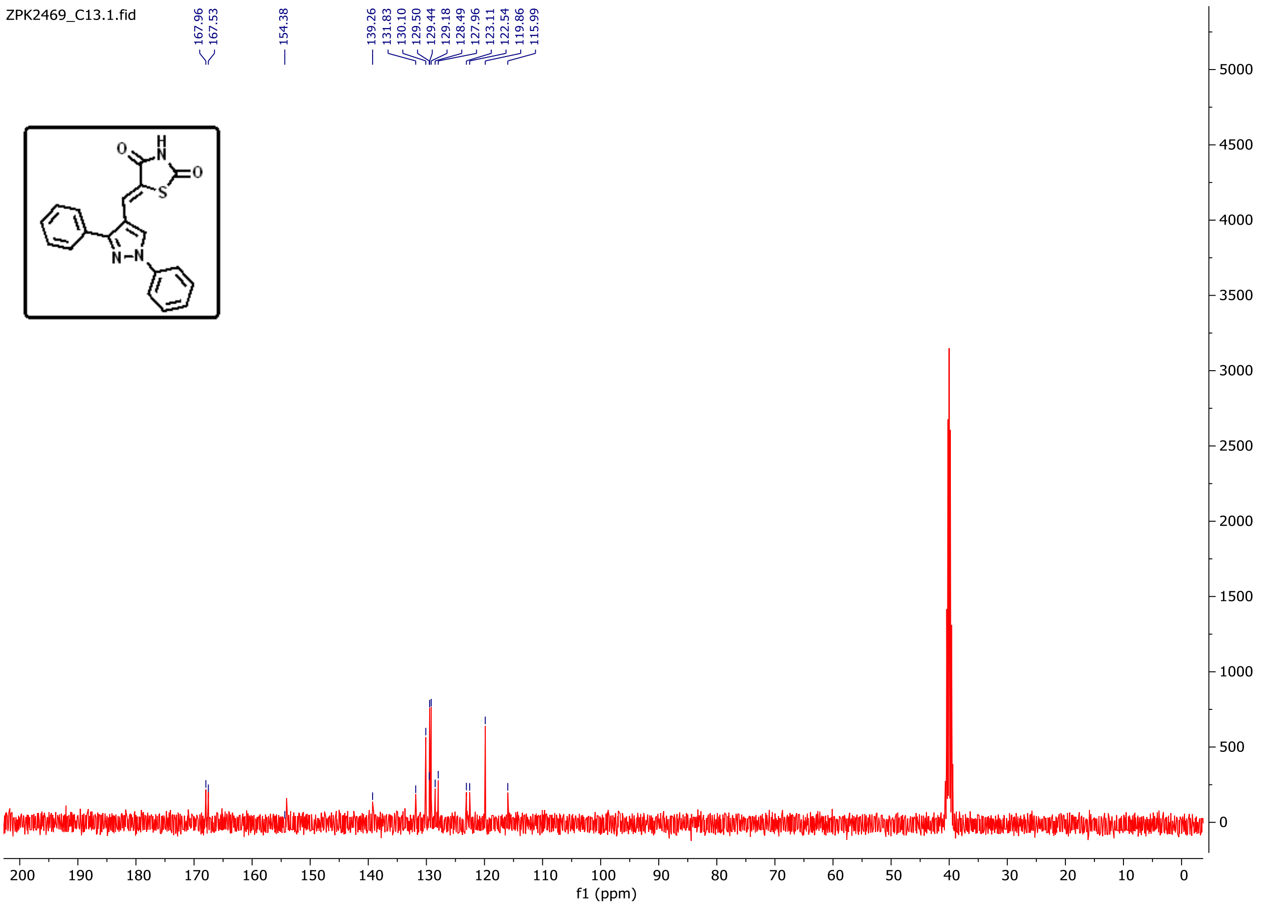

Figure S2. ^{13}C NMR spectrum of compound **iii** (Les-6490).

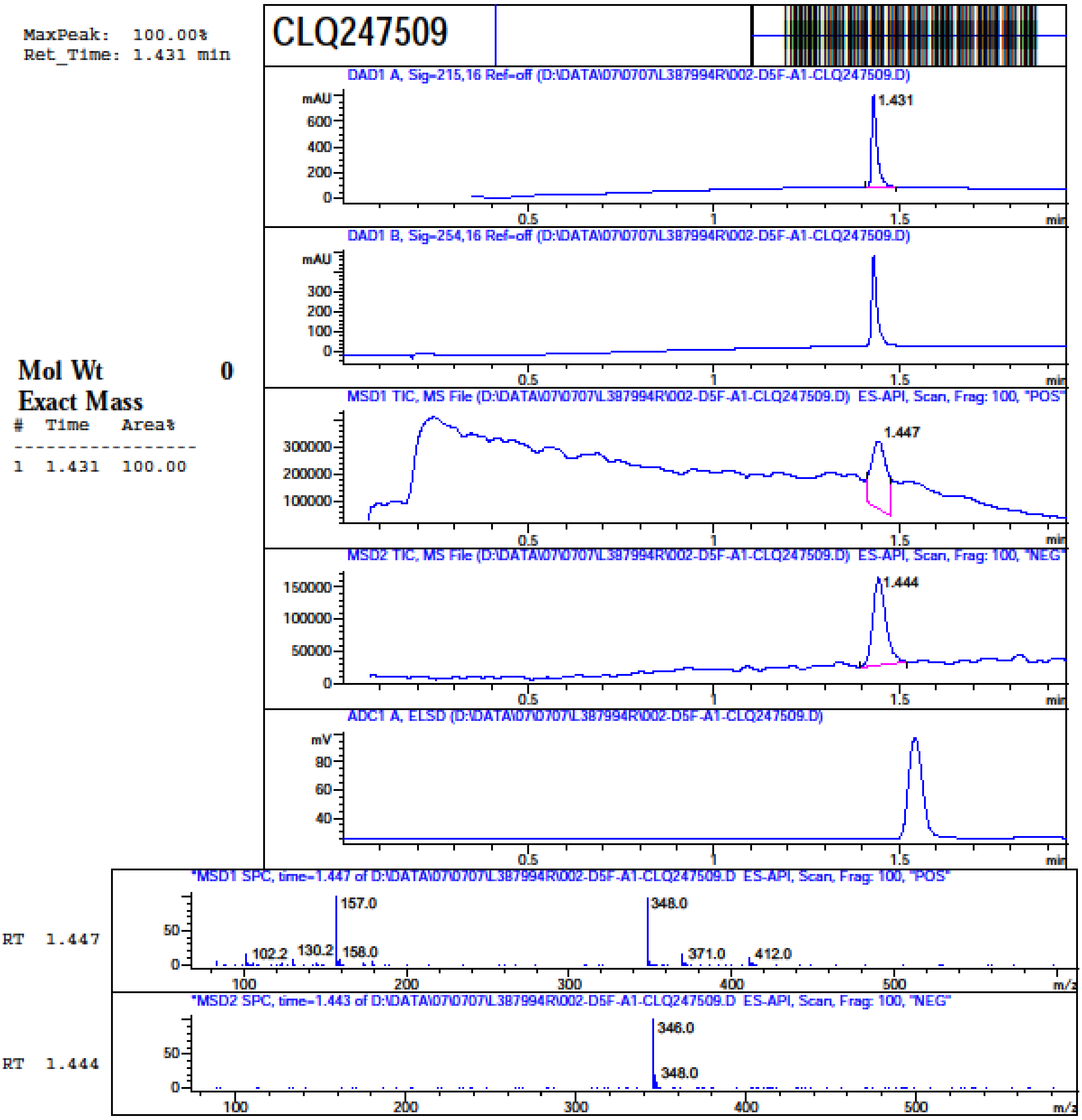

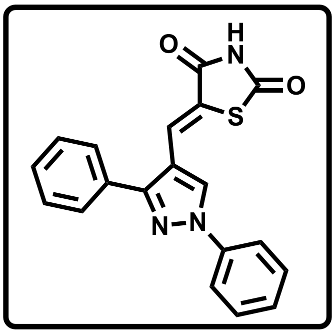

Molecular Weight: 347,39

Figure S3. LC-MS spectrum of compound **iii** (Les-6490).

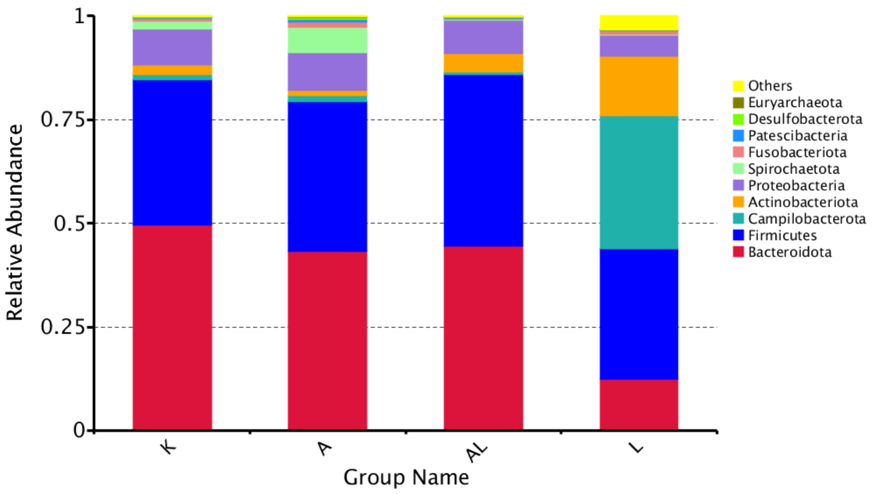

Figure S4. Comparative Taxonomic Relative Abundance at the Phylum Level Across Different Groups (K, A, AL, L). This bar chart illustrates the distribution and relative abundance of different microbial taxa at the phylum level, highlighting dominant phyla in the fecal microbiota of groups K (Control without adjuvant and Compound), A (Adjuvant), AL (Adjuvant + compound), and L (Compound without adjuvant). It underscores variations in microbial composition between these groups.

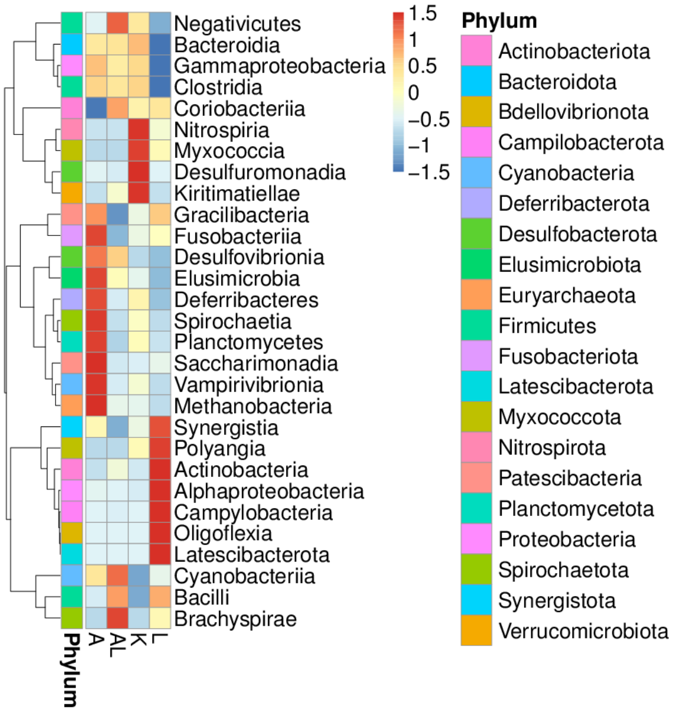

Figure S5. Taxonomic Abundance Cluster Heatmap at Cluster' C'. This heatmap presents clustering of taxonomic abundance at a specific cluster level, denoting significant variations in microbial populations across different groups: K (Control without adjuvant and Compound), A (Adjuvant), AL (Adjuvant + compound), and L (Compound without adjuvant). The color intensity in each cell reflects the abundance level of taxonomic entities, facilitating a visual comparison of microbial community structures among the groups.

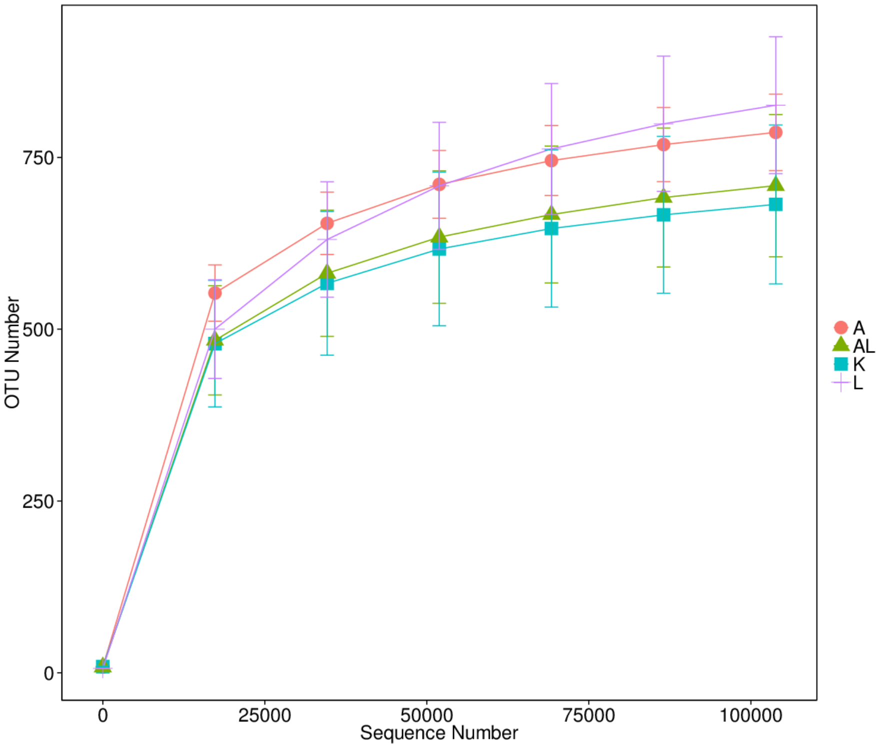

Figure S6. Biodiversity Curve Across Different Study Groups. This figure presents the biodiversity curves, showcasing species richness and diversity within the microbial communities of each group, including K (Control without adjuvant and Compound), A (Adjuvant), AL (Adjuvant + compound), and L (Compound without adjuvant).

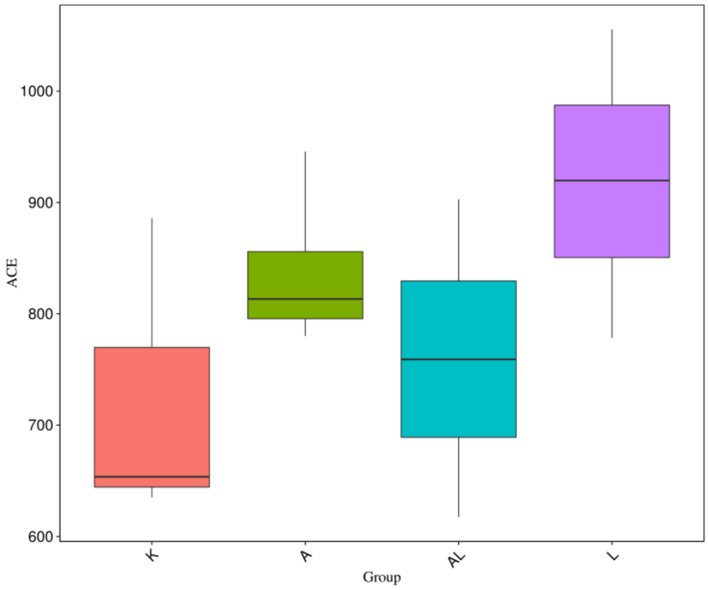

Figure S7. Box Plot of the Difference in Observed Species Across Study Groups. This box plot illustrates variations in the observed species count among each group: K (Control without adjuvant and Compound), A (Adjuvant), AL (Adjuvant + compound), and L (Compound without adjuvant). Statistical analysis using the Wilcoxon Signed Rank test for paired samples: p-value=0.4336, Z=2.500 between A and AL groups, p-value=0.3259 Z=3.416 between K and A groups.

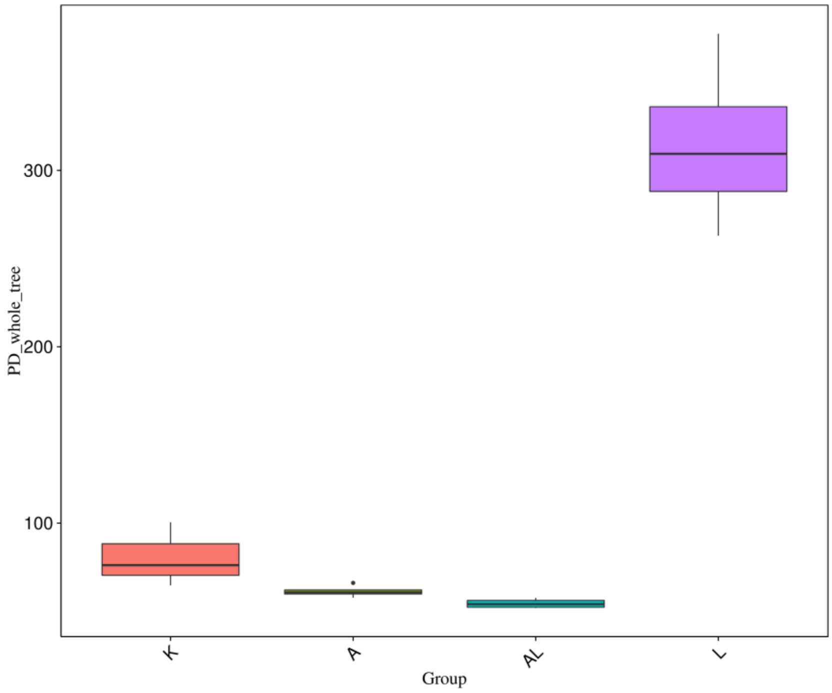

Figure S8. Box Plot of Phylogenetic Diversity (PD) Whole Tree Across Groups. This plot illustrates the phylogenetic diversity within the microbial communities of groups K (Control without adjuvant and Compound), A (Adjuvant), and AL (Adjuvant + compound). Statistical analysis using the Wilcoxon Signed Rank test for paired samples: A–AL (Z=4.250000 p-value=0.0017), A–K (Z=-2.916667 p-value=0.0239), AL–K (Z=7.166667 p-value=0.0000).

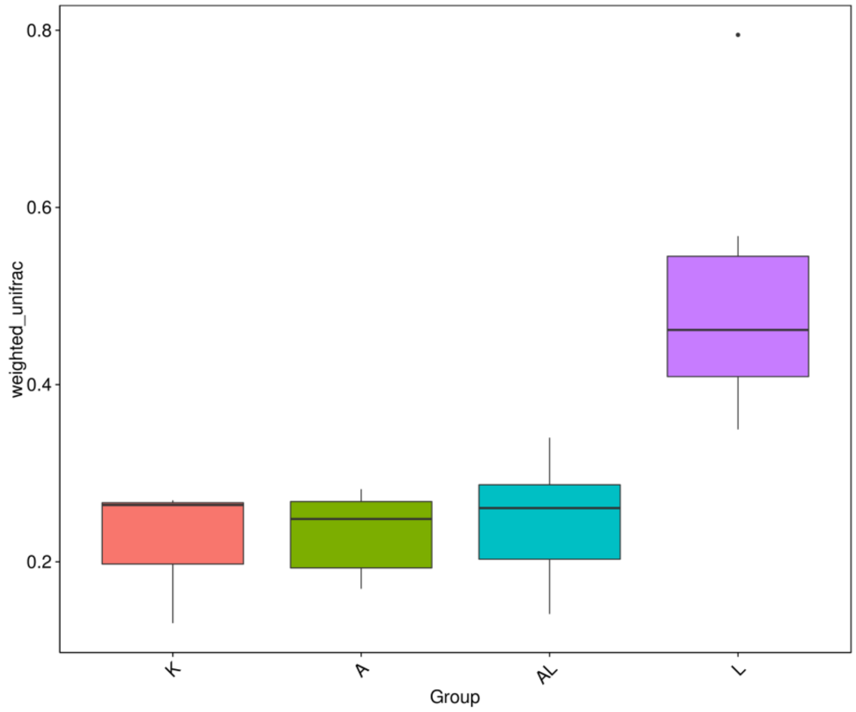

Figure S9. Box Plot Based on Unweighted UniFrac Distance Across Groups. This plot displays the microbial community dissimilarities among groups K (Control without adjuvant and Compound), A (Adjuvant), AL (Adjuvant + compound), and L (Compound without adjuvant) using the Unweighted UniFrac distance metric. Wilcoxon Signed Rank test for paired samples: p-value > 0.05 between A-AL, A-K, K-AL groups, p-value < 0.05 for A-L, K-L, and Al-L groups.

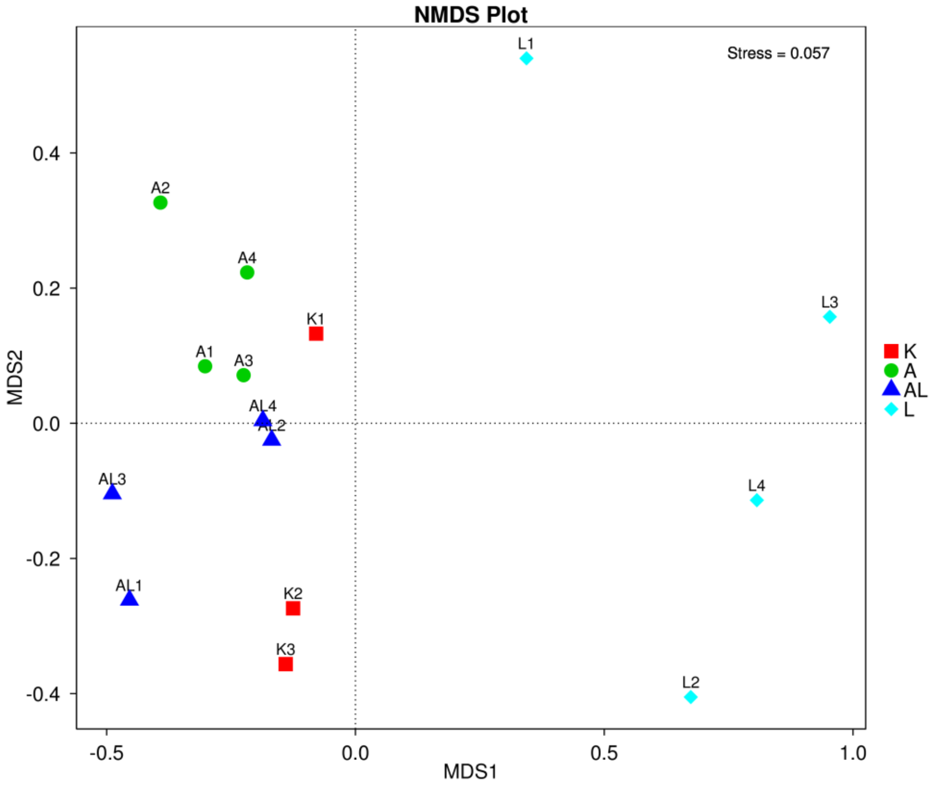

Figure S10. Non-Metric Multidimensional Scaling (NMDS) Analysis of the 4 Groups. This NMDS plot visualizes the similarities and dissimilarities in microbial community structures among the four study groups: K (Control without adjuvant and Compound), A (Adjuvant), AL (Adjuvant + compound), and L (Compound without adjuvant). Points' spatial distribution indicates the relative ecological distances between the groups, highlighting their distinct microbial profiles.

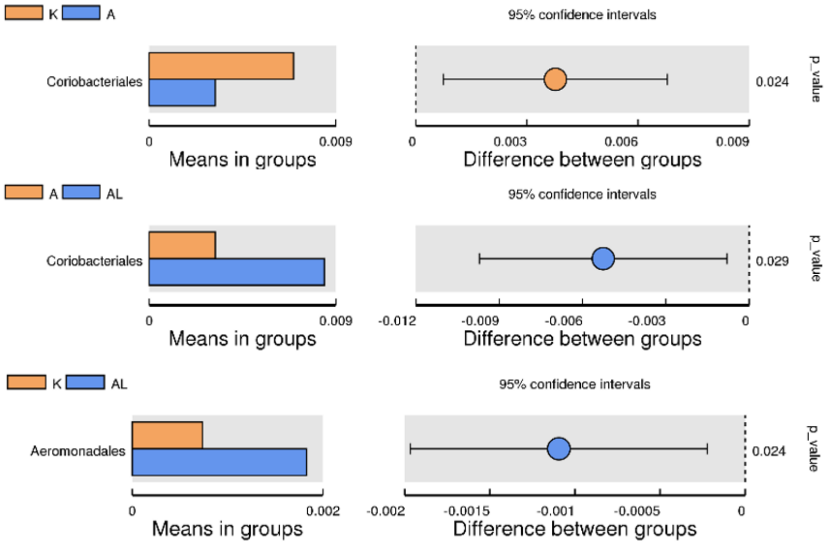

Figure S11. Between-group T-test Analysis (Orders *Coriobacteriales*, *Aeromonadales*). This figure presents T-test analyses results comparing the abundance of microbial orders *Coriobacteriales* and *Aeromonadales* across the four study groups: K (Control without adjuvant and Compound), A (Adjuvant), AL (Adjuvant + compound), and L (Compound without adjuvant).

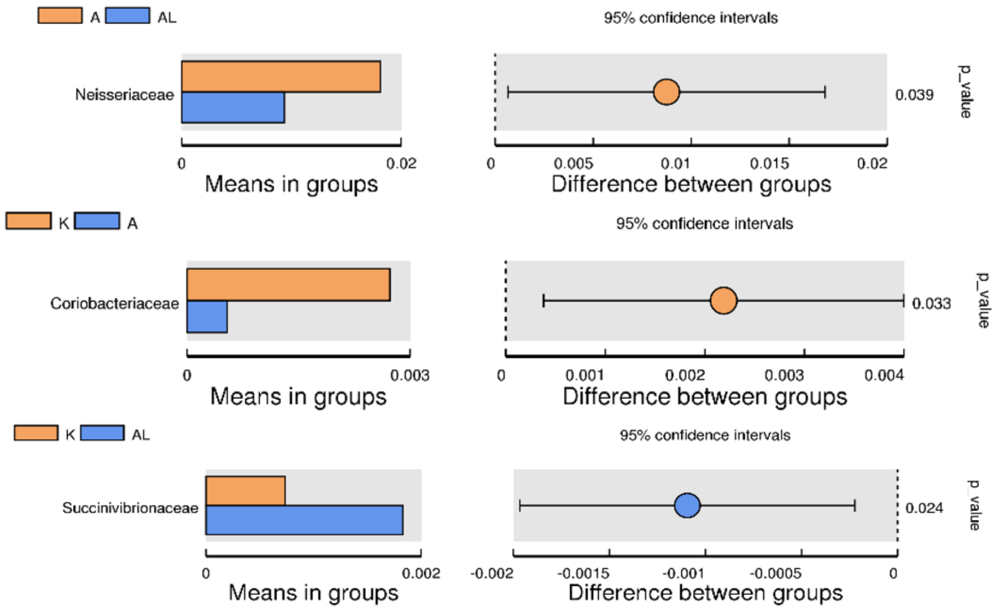

Figure S12. Between-group T-test Analysis (Families *Neisseriaceae, Coriobacteriaceae, Succivibrionaceae*) Among Groups K (Control without adjuvant and Compound), A (Adjuvant), AL (Adjuvant + compound), and L (Compound without adjuvant). The analysis aims to identify significant differences in these families' abundance, reflecting their varied responses to treatment conditions.

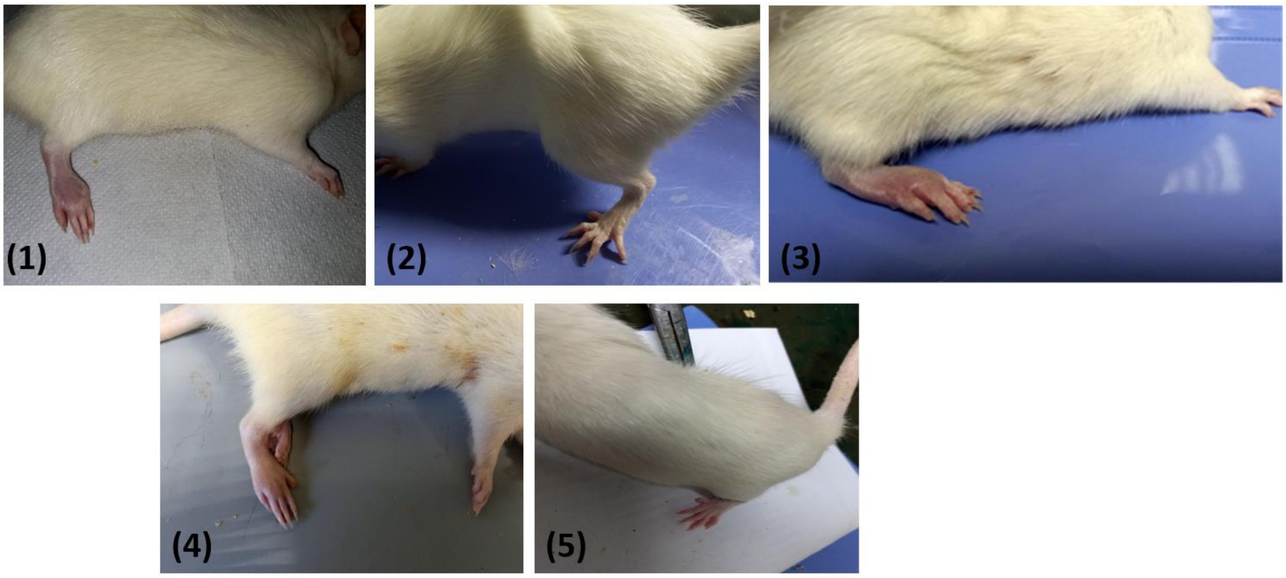

Figure S13. Photos of Rat Paws at Different Experimental Stages. **(1)** The photo of the rat paw after the introduction of Freund's adjuvant (FA) shows signs of inflammation, swelling, and redness. **(2)** Group K (control, intact animals, without induced inflammation). **(3)** Group A, animals with Freund's Adjuvant-induced inflammation on day 13, showing severe swelling with limitation of passive mobility, inability to bend the ankle to the lower leg, erythema; the circumference of the affected area of the foot is 9.4 mm. **(4)** Group A+L, animals with induced inflammation treated with Les-6490, showing swelling and erythema from the ankle to the metatarsal bone, reduction of the affected area of the foot to 7.8 mm on day 13. **(5)** Group L, animals that received the compound Les-6490 without induced inflammation, showing a normal foot on day 13 of the experiment.
